# Ask the Locals: A Community-Informed Analysis of Perceived Marine Environment Quality Over Time in Palawan, Philippines

**DOI:** 10.3389/fpsyg.2021.661810

**Published:** 2021-08-10

**Authors:** Joel D. C. Sumeldan, Isabell Richter, Arlene L. Avillanosa, Hernando P. Bacosa, Lota A. Creencia, Sabine Pahl

**Affiliations:** ^1^College of Fisheries and Aquatic Sciences, Western Philippines University, Puerto Princesa City, Philippines; ^2^School of Psychology, University of Plymouth, Plymouth, United Kingdom; ^3^Department of Psychology, Norwegian University of Science and Technology (NTNU), Trondheim, Norway; ^4^Department of Biological Sciences, Mindanao State University-Iligan Institute of Technology, Iligan, Philippines; ^5^Urban and Environmental Psychology Group, Institute for the Psychology of Cognition, Emotion and Methods, Faculty of Psychology, University of Vienna, Vienna, Austria

**Keywords:** future perception, community perceptions, habitats, sustainable development, local management, provisioning ecosystem services, marine issues

## Abstract

Despite the potentially huge contributions that coastal communities might make in marine resource management and sustainability, their participation in such efforts have only been recognized recently, particularly in Southeast Asia. Involving community perceptions can offer new insights for policy makers and resource managers and can elicit strong commitment and support from the communities themselves. This article aims to understand the perceptions of coastal communities of local environmental issues, specifically how these have developed over time, to understand the expectations and perceptions of trends. Sixteen marine environmental issues were identified during stakeholder meetings in Palawan, Philippines. A co-developed survey was administered to 431 respondents from coastal communities in two municipalities (Taytay and Aborlan) and in the city of Puerto Princesa in Palawan. The results show variation in the perceptions and expectations across issues. We find that communities expect positive trends for mangrove coverage, beach tree cover, and seagrass coverage as well as for seaweed farming and quality of drinking water. The amount of plastic litter, wild fish and shellfish, and the severity of sewage pollution are perceived to get slightly worse. The aquaculture sector is expected to remain unchanged in the future as it had been in the past. We also find significant differences in how people from different areas of residence perceive their marine environment. In the discussion, we mapped these different community perceptions on existing policies and their implementation. We further recommend how community perceptions can be integrated into resource management and policy making in the future.

## Introduction

Over three billion people directly depend on marine and coastal biodiversity for their livelihoods and on marine resources as their main source of protein (OECD, 2020). Especially in developing, coastal countries and in island states, the ocean plays a key role in economic survival. However, marine resources across the globe are under pressure as a result of continuous and increasing human exploitation (Halpern et al., 2012). The United Nations have dedicated one of the Sustainable Development Goals (SDGs) adopted in 2015 to “conserve and sustainably use of the oceans, seas and marine resources for sustainable development” [United Nations General Assembly (2014), SDG 14, Life Below Water]. Such a goal requires not only global commitment, but also localized strategies. The success of these localized strategies for sustainable management depends on a comprehensive assessment of the environment to tailor the approach, and the acceptation of these strategies by the population. Thorough assessments of the respective areas including its main assets and pressures can be a lengthy and costly process, requiring comprehensive scientific data, biological measurements, and literature (Gebremedhin et al., 2018). The importance of the knowledge of local communities is sometimes disregarded, leading to conflicts between policy makers and communities (Bennett and Dearden, 2014). This article is set in the context of increasing calls for the efficient use of marine resource management by considering the community perceptions.

Actively engaging with local communities could be a way of achieving both, gaining crucial insights and reaching acceptance. Local communities can provide unique information about the state of marine ecosystems. They not only directly depend on their local marine environment, but are also exposed to its changes over time, sometimes over the course of their whole life, which can provide unique local ecological knowledge to interested scientists (Pauly, 1995). They also gather information about the past from family members and elders and might discuss with other community members about the future. Data on temporal perceptions of change are rarely collected, even though past experiences and future expectations guide community behavior and responses to regulation and management options. Feeding such community insights into management of local areas is likely to lead to more compliance (Sherriff et al., 2019).

Management strategies typically rely on data from the natural sciences which may be challenging and costly to collect (Fenner, 2012), with sometimes insufficient quality (Bjørkan and Rybråten, 2019). Given the time and financial constraint a conventional scientific sampling could incur, particularly in the subtropical and developing countries, availing community participation through their local knowledge and perception in conservation efforts has lately demonstrated promising results to mitigate these problems, while this approach is obviously not a replacement for obtaining scientific data (Drew, 2005; Silvano et al., 2006; Silvano and Begossi, 2010; Leite and Gasalla, 2013; Turvey et al., 2013). Local knowledge can help the decision makers to develop locally feasible solutions (Leite and Gasalla, 2013). Integrating local knowledge in resource management planning seems to promote co-management schemes between resource users and managers (Silvano and Begossi, 2005; Silvano et al., 2006; Hamilton et al., 2012; Abecasis et al., 2013; Leite and Gasalla, 2013; Bulengela et al., 2019). This is because of information on issues, such as fish populations (Aswani and Hamilton, 2004), fish diet (Silvano and Begossi, 2010) and fish reproduction (Silvano et al., 2006), and freshwater cetacean population (Leite and Gasalla, 2013), which were commonly gathered using methods from the natural sciences, can also be gathered through local knowledge. Building on the knowledge and perceptions of the people can provide unique insights and thereby facilitate tailor-made governance (Christie et al., 2003; Akpabio, 2011). Local communities can provide information beyond biological parameters in the form of priorities of provisioning ecosystem services, dynamics between people and environments as well as traditions and norms. Local ecological knowledge is a prerequisite for local, context-specific solutions that help to cope with changing environments and facilitate climate change mitigation and adaptation (Mistry and Berardi, 2016). If these two types of information are cross-verified and combined, we could create a comprehensive knowledgebase for more adaptive and effective resource management (Silvano et al., 2006; Butler et al., 2012; Hamilton et al., 2012).

Moreover, a perception-based approach could be used to clear misunderstandings or explain divergence between resource managers and users (Bacher et al., 2014). It can help to understand and mediate conflicts between people holding different opinions and attitudes (Bacher et al., 2014) and overcome barriers of communication among stakeholders which can ultimately support the achievement of resource management goals (Nursey-Bray et al., 2012). It could give an indication to managers on how to communicate with the local population about management strategies though disagreement cannot always be avoided (Leleu et al., 2012; Bjørkan and Rybråten, 2019). The acknowledgment of community perceptions of their ecosystems can facilitate successful marine resource management (Thaman et al., 2016), create a spirit of co-creation, and establish trust and acceptance (Maurstad, 2002). In many cases, top-down governance models have turned out to be problematic due to non-acceptance from local communities resulting in poaching, sabotage, and other illegal activities (Lockwood, 2010). The transition of community-based management to centralized national management of Apo Island in the Philippines is only an example in which the attempt of top-down sustainable management failed because of the exclusion of the perception of the local people (Hind et al., 2010). Therefore, understanding the perceptions of the marine resource users and adapting communication strategies to these are key policies for acceptance and support, and for sustainable behavior change (Moser, 2014; de Vries, 2020).

Perceptions of local communities are still seldom included in management plans and if they are included, they are mostly acquired through a community representative, with a risk that this person might not be able to represent a variety of opinions, leaving other perspectives and minorities unheard (Wever et al., 2012; Kitolelei and Sato, 2016). Even if perceptions from local communities could be collected, its integration in resource management is still difficult (Hind, 2015). This might be due to lack of a management framework that includes perceptions in complex system of sustainable development (Ostrom, 2009). While it is true that including all people in the management discussions would be impractical, relying on a representative to represent a community might still be insufficient; thus, a suggestion of having perception expert has been proposed by Beyerl et al. (2016).

Most attempts to involve local communities in sustainable resource management strategy development retrieve insights from qualitative interviews and focus groups asking about current resource management and conflicts (Hens, 2006; Vodouhê et al., 2010; Akpabio, 2011; Aretano et al., 2013). Furthermore, most research on community involvement is conducted in the Global North while research is still scarce in the Global South. Hence, especially in the Global South, gathering and integrating community perception data could help policy makers implement regulations and make appropriate marine resource management decisions (Allison et al., 2012; Leite and Gasalla, 2013).

### Perceptions With a Temporal Dimension

Very little is known about community perceptions of changes over time, especially when applying a human time horizon of a maximum of one generation (Pahl et al., 2014; Fincher et al., 2015).

Given the familiarity of communities with their home, it is likely that, beyond being able to report how their environment is at that moment, they have a reasonable picture of how their environment developed in the past but also how they expect their environment to look like in the future. These perceptions are not only relevant as a source of information but also because they determine if sustainable management practices are accepted. Past perceptions are relevant for sustainable resource management as they allow for mapping the success of local regulations that are already in place with the perceptions of the people. Perceived changes from the past to the present can give an indication of areas that are more salient to communities than others and areas that might need more attention. Different outlooks on the future activate different psychological and behavioral processes. Anticipated *negative* events or consequences have been found to evoke intentions for pro-environmental behavior (Sanna, 1996; Carrus et al., 2008; Pestridge, 2017). Several studies point toward fear or worries related to the future being a principal antecedent for risk prevention behavior (LaTour and Tanner, 2003; Dillard and Anderson, 2004). On the other hand, it was found that the more *positive* expectations people have about the future, the more preparation actions they undertake (Kornadt et al., 2015). Imagining a positive future can increase the likelihood of this future to become reality if people believe in their success, i.e., have a high level of self-efficacy (Oettingen et al., 2009; Adriaanse et al., 2010; Kappes et al., 2012). Beyond the behavioral effects, positive expectations of the future can evoke higher resilience and psychological well-being (Keyfitz et al., 2013; Stoknes, 2015), whereas negative expectations for the future, for example, in terms of the effects of climate change, can result in poor mental health (Ogunbode et al., 2021). In a study exposing people to positive, negative, and neutral future scenarios, researchers found that the mere fact that people are engaged with their future increases their intentions to perform more sustainable actions (Richter et al., Under Review). Yet, it has not clearly been established if positive anticipations or negative anticipations are more effective in driving intentions for behavior change, but it seems that being aware of future benefits inspires to take sustainable actions across nations (Bain et al., 2015). For sustainable resource management, both positive and negative expectations and their resulting effects are relevant for tailoring management strategies.

### Policies and Community Perceptions on Palawan Island

Palawan Island is one of two biosphere reserves in the Philippines. Its sustainable development is generally governed by the Palawan Council for Sustainable Development (PCSD), the Department of Environment and Natural Resources (DENR), together with the local government from the Barangay, Municipality and Province. In the last couple of years, Palawan has been gaining momentum in sustainable landscape and seascape development. One of the strongest laws made in this area is the Strategic Environmental Plan (SEP) for Palawan Act of 1992, serving as a framework to guide the government units and agencies in the formulation and implementation of programs for natural resource management. It is through this law, the PCSD has been established and given the power to create policy, monitor, coordinate, and implement rules. The office also promotes co-management scheme and information campaign along with enforcement measures. However, despite such effort, illegal activities in the marine and coastal areas of Palawan still occur. These include illicit cutting of mangrove, illegal fishing, and illegal trade of wildlife. Recognizing the importance of a science-based management approach, the council, along with other government and non-government agencies, continues to welcome studies on policy making toward sustainable development. So far, most research projects that were conducted to support resource management focus on physical and biological aspects of the environment with its living and non-living components, but are limited in terms of the human dimension. Local perceptions have been explored rarely in this province, but some examples exist for Marine Protected Areas (MPAs) and natural parks. Understanding the perspectives of the local people is important and in line with the acknowledgment of the area as “Man and Biosphere” reserve.

In a small number of studies, perceptions of the importance of natural resources have been investigated. Jalani (2012) assessed how communities in Sabang, Puerto Princesa City, think about ecotourism in order to frame communication approaches accordingly. Garces et al. (2013) used local community perceptions from people living in northern Palawan as one of the indicators of management effectiveness of three marine protected areas, concluding that bringing both, natural science-derived information and community perceptions together, provides useful information for the governance as compared to both information sources on their own. Pana and Su (2012) investigated how perceptions around climate change can influence climate change- related actions, finding that respondents who did not perceive climate change as a threat were less likely to implement actions. Expanding this work in other parts of Palawan on different topics (e.g., water quality, shellfish resources, and aquaculture development) and with an added temporal dimension could help in understanding the local community responses when new policies are adopted.

In the present study, we therefore explore the perceptions of local people regarding past and future marine environment quality in three different areas in Palawan, and discuss implications for sustainable marine resource management. These insights can later potentially feed into reformulation or improved implementation of existing laws as well as the development of new regulations and programs. More specifically, this study aims to (1) identify the main issues local communities are facing in relation to the marine environment; (2) assess the present and future perceptions of coastal communities on these marine environmental issues in three municipalities of Palawan; (3) explore how the perceptions of people on marine environmental issues in Palawan over time vary in relation to different demographics, including the area of residence.

By doing so, we provide data of community perceptions as a guide for assessing their potential level of cooperation, commitment, and support for marine management plans and policies. This could also serve as baseline data for potential future studies. Whilst this knowledge might mostly be relevant for local governance in Palawan, the same principles of data collection can be applied for other coastal regions in the Philippines that are in need of integrated marine management.

## Materials and Methods

### Study Area

The study was conducted on the island of Palawan, located at the southwestern side of the Philippine archipelago. Palawan is the largest province among the 81 provinces of the Philippines. The province has a total area of 1,489,626 ha, a coastline of about 2,000 km and is composed of 1,768 islands housing a unique marine flora and fauna, making it one of the largest areas in marine biodiversity in the Philippines (PCSDS, 2015). Although the island is large, only a relatively small portion is considered habitable due to its topography. Tall mountain ranges and hills make up the 63% of the total land areas which are located at the center of the main island and divide it into two distinct areas, the east and the west coast (PCSDS, 2015). The remaining 37% which are relatively flat to gentle slopes are generally located near the coast (PCSDS, 2015). Having these mountainous areas, Palawan people rely primarily on forest as their principal resource (source of rattan and almaciga resin); forest covers almost 46% of the total land area (PCSDS, 2015). Another major economic activity on the island is agriculture, utilizing both flat lands and mountainous areas, which can lead to conflict with forest use. Together with mineral and ore extraction, as well as population growth, natural resources are increasingly declining and therefore posing a threat to the future of Palawan. Estimation in 2015 shows more than 1.1 million inhabitants in Palawan, with an annual population growth of 1.84% (Philippine Statistics Authority [PSA], 2020). Compared to the neighboring provinces, Palawan is one of the least populated areas with 58 persons per square kilometer, excluding the Puerto Princesa City with 107 persons per square kilometer (Philippine Statistics Authority [PSA], 2016). Although the agricultural sector still offers the largest employment, there is a gradual shift toward the service sector.

As an island, coastal and marine resources, such as fisheries, coral reefs, mangroves, and seagrass beds also provide a major source of livelihood to the people of Palawan, particularly to those living near the coastal areas. The province contributes more than 120,000 metric tons of fish to the whole Philippine marine fisheries production, making it one of the top marine fisheries producing provinces in the country (Philippine Statistics Authority [PSA], 2020). However, as in other parts of the world, decreasing trends have been observed for fish populations (PCSDS, 2015). The same goes for seagrass and coral cover. Moreover, its mangrove area is the largest in the Philippines, covering approximately 534 km^2^ ha in 2016 and future trends are currently uncertain (Bunting et al., 2018).

### Sample

**FIGURE 1:**
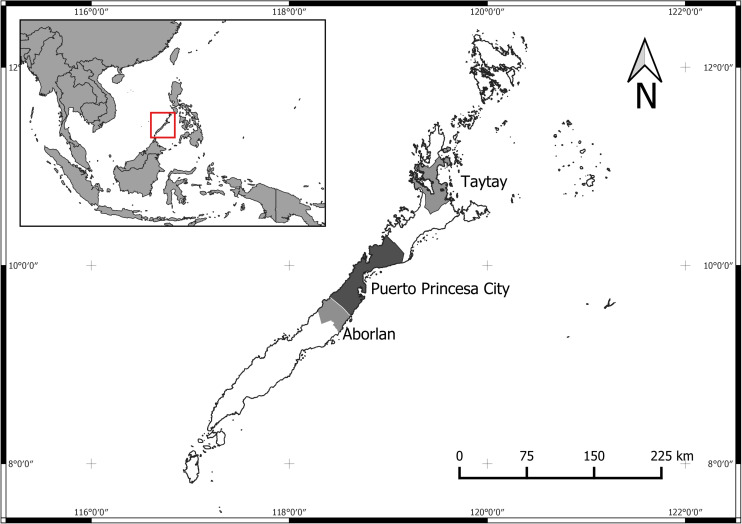
Map showing the province of Palawan, Philippines, and the study areas – the municipalities of Taytay, Aborlan, and Puerto Princesa City. The red square highlights the Palawan island location relative to Southeast Asia.

From the Palawan province, three municipalities were selected as study areas: Aborlan, Taytay, and Puerto Princesa City (Figure 1). In these municipalities, eight (across Taytay and Puerto Princesa City) and two (across Aborlan) small geographical areas (barangays, the smallest political unit in the Philippines) were selected for the data collection (10 in total). Barangays located along the coastline were selected for the purpose of this study to assure that respondents will have sufficient knowledge on the marine environment. In addition, some of our respondents were also among those who are beneficiaries of the livelihood program of the government, particularly on seaweed farming. As such, they receive seaweed propagules and implements for free.

A total of 431 respondents, each representing one household from a total of 10 barangays in three municipalities, participated in the survey. This estimate was based on the ten percent (10%) of approximate total number of households provided by the Barangay. All respondents live within <1 km from the marine coast. Selection of municipalities and barangays was primarily based on their history in fisheries and coastal resource management plus the existing academic-stakeholder relationship and their willingness to participate in the research.

The age of respondents ranged from 19 to 90 years old, with most respondents being concentrated around the 30–49 age bracket (Table 1) which contrasts with the general population of Palawan, where the majority are in the 18–29 years old bracket (Philippine Statistics Authority [PSA], 2017). The average income was estimated at USD 175.75 (PHP 8,459) per month. In relation to the whole Philippine population, the majority of the respondents (56.4%) were considered poor with an income of less than USD 164.02 (PHP 7,980) per month (Albert et al., 2015). The sample consisted of 59.8% female as compared to 48% of the general population of Palawan. Only few of the respondents (13.2%) had completed tertiary education, while most of them had completed primary education and/or secondary education which coincides with the general population of Palawan, in which the majority had completed high school and elementary education (47.3% and 39.5%, respectively). Religion among the respondents was predominantly Roman Catholic (80.8%) while ethnic group was dominated by Visaya (43.1%), followed by Cuyono (19.7%).

**TABLE 1:** Demographic characteristics of the respondents (*N* = 431).

Individual-level variables	*N*	Percent	Mean	SD
**Age**	427	100	44.9	13.6
Group 1 (18–29)	61	14.3		
Group 2 (30–39)	102	23.9		
Group 3 (40–49)	114	26.7		
Group 4 (50–59)	84	19.7		
Group 5 (60–90)	66	15.5		
Missing data (*n*)	4			
**Gender**				
Male	173	40.2		
Female	257	59.8		
Missing data (*n*)	1			
**Ethnic group**				
Cuyono	84	19.7		
Ilonggo	26	6.1		
Others	66	15.5		
Tagalog	53	12.4		
Tagbanua	14	3.3		
Visaya	184	43.1		
Missing data (*n*)	4			
**Monthly income (USD)**	411	100	175.8	185.5
Poor (<164.02)	232	56.4		
Low income (164.02–328.04)	146	35.5		
Lower middle income (328.04–656.09)	27	6.6		
Middle income (656.09–1,640.26)	4	1.0		
Upper middle income (1,640.26–2,460.40)	2	0.5		
Upper income (2,460.40–3,280.53)	0	0.0		
Rich (>3,280.53)	0	0.0		
Missing data (*n*)	20			
**Education**				
Primary(finished and not finished)	168	39.5		
Secondary (finished and not finished)	201	47.3		
Tertiary(finished and not finished)	56	13.2		
Missing data (*n*)	6			
**Religion**				
Roman Catholic	345	80.8		
Baptist Church	17	4.0		
Born Again Christian	22	5.2		
Iglesia Ni Cristo	9	2.1		
Islam	2	0.5		
Others	32	7.5		
Missing data (*n*)	4			
**Location**				
Aborlan	75	17.4		
Taytay	187	39.2		
Puerto Princesa	169	43.4		
**Occupation**				
Aquaculture (caged fish/fin-fish)	17	4.3		
Crabbing/shrimping	20	5.1		
Fishing	300	76.7		
Post-harvest activities	7	1.8		
Seaweed farming	44	11.3		
Taking tourists on boats, etc.	3	0.8		
Missing data	40			

### Focus Group Discussions

As a part of the early development of the survey, three stakeholder group meetings were convened. The first was in Puerto Princesa City at the center of the island, the second in Aborlan in the south, and the third in Taytay in the north. The meetings involved 28, 30, and 29 people in Aborlan, Taytay, and Puerto Princesa City, respectively (Table 2) with local stakeholders and local and international academics. Local stakeholders included representatives of the fishing communities, local health care workers, social workers, local environmental officers, local agriculture and fisheries officers, local legislation members, and local community leaders (e.g., Barangay Captains – men and women). In all workshops, there were more men than women attendees ranging from 60 to 74%.

**TABLE 2:** Representation of participants who attended the workshops.

Representative	Aborlan		Puerto Princesa City		Taytay	
	Male	Female	Male	Female	Male	Female
Local Legislation Member		2			1	2
Local Environmental Officer	1		3		1	1
Local Government Administration Officer	1				1	
Local Social Worker	1					
Local Health Care Workers		1		1	2	1
Local Government Planning and Development Officer	1		1		1	
Fishing Community Representative		1			3	
Local Agriculture/Aquaculture Officer	1		1		1	1
Local Government Head	1					
International Academe	2	2	4	2	3	2
Local Academe	9	5	12	5	6	3
Local Community Representative					1	
Total	17	11	21	8	20	10

These workshops focused on three questions: (a) What are the main challenges in your area or field of expertise? (b) What are your top priorities in terms of health and well-being in your area or field of expertise? and (c) What are your main hopes/aspiration in the future in your area or field of expertise? In each workshop, the group was broken up into smaller subgroups of 4–6 people to answer each of the three key questions. Since the focus group discussions involved international academics (University of Exeter and University of Plymouth) and since many of the local stakeholders could not speak English well, group discussions were undertaken in the local language with local academics. Discussion outcomes were then noted on a poster paper and translated back to English during the plenary group discussions. Each workshop in these two areas lasted up to 3 h in the morning, from 9 a.m. until lunch. In one workshop (Puerto Princesa City), additional large maps of the two local areas were used and participants were encouraged to draw on them about the things that they thought were important to consider for both areas (Supplementary Figure 1). For instance, one group drew the site of new aquaculture pens and seaweed farming lines, another showed locations where the mangroves had been extensively cut back; one group drew the location of a new poultry farm that they said was polluting the local water supply, and another the site of slash-and-burn forest clearance. These maps were then used as a guide to frame discussions for the other two workshops (Aborlan and Taytay).

The maps and associated texts from the three stakeholder meetings were then discussed at another workshop (Puerto Princesa) held between the local and international academics, in order to group emerging environmental issues and sketch out a structure and content for the survey, strictly following co-creation principles. Issues were framed in-line with the ecosystems-enriched Drivers, Pressures, State, Exposure, Effects, Actions (eDPSEEA) framework which considers human activity and health along with complex interaction with the environment (Reis et al., 2015). In particular, six research questionswere formulated for the survey, of which only the first two is relevantin the context of the present article on temporal perceptions: (a1) Compared to 10 years ago (2009) would you say the state of the following features in Palawan is better, worse or the same?; (a2) Compared to now, how do you see the state of the following features in Palawan in the future in 10 years’ time (e.g., 2029)?; (b) How have the following issues changed the quality of the local coastal environment (for better or worse)?; (c) In the last 7 days, how often have you done the following activities?; (d) In the last month (4 weeks), have you experienced any of the following health outcomes as a result of spending time in/on/around the water?; and (e) How important do you think each of the following factors is for local health and well-being?. Since this study focused on the state of the marine environment, most issues and thoughts were excluded herein but included in the whole survey for other studies which corresponds to other parts of the eDPSEEA framework. Issues found across the study areas which were included in the state of the marine environment section were the following: conflicting land and water use (e.g., mangrove conversion to residential, zone for mariculture areas), illegal fishing, overfishing, waste management, and the use of chemicals in farming. Combining these issues with resources drawn in maps, such as mussel farm, coral reef, sea grass, mangroves, human settlements, pearl farm, dump site, quarry, seaweed, and rice fields, the sections were then populated with such issues. With further workshops and consultation from local experts, these issues were further finalized into 16 issues categorized into three groups namely, resources, habitat, and water quality. Survey questions were then formulated revolving around the 16 issues which were then included in the final survey (Tables 3, 4).

**TABLE 3:** Questions and distribution of answers among respondents for present perceptions with the following response options: much worse = −3, slightly worse = −2, worse = −1, the same = 0, better = 1, slightly better = 2, much better = 3, and prefer not to answer = 99.

Compared to 10 years ago (2009) would you say the state of the following features in Palawan is better, worse the same?	−3	−2	−1	0	1	2	3	99	Total
Amount (number) of wild fish, diversity of fish types	37	104	117	56	40	35	31	11	431
Amount of wild shellfish, diversity of shellfish types	23	68	88	99	38	42	29	44	431
Amount of fish aquaculture (e.g., fish cages)	17	22	53	53	28	30	15	213	431
Amount of shellfish aquaculture (e.g., mussel lines)	8	6	22	22	12	21	14	326	431
Amount of seaweed farming	10	10	27	31	20	34	20	279	431
Quality of coral reefs, diversity of coral types	25	62	88	65	45	51	36	59	431
Seagrass coverage, number of seagrass species	7	27	42	149	45	56	48	57	431
Mangrove coverage, diversity of mangrove types	11	30	65	93	63	78	66	25	431
Other beach tree cover	3	24	57	140	70	61	51	25	431
Amount of farming pesticides/fertilizers in the water	10	12	63	76	34	20	11	205	431
Amount/concentration of sewage in the water	18	44	107	89	41	31	17	84	431
The amount of plastics/rubbish in the water	35	62	101	85	50	53	31	14	431
The color and smell of the sea water	12	45	68	196	36	43	18	13	431
The taste of fish/shellfish from these waters		6	27	316	20	37	15	10	431
The frequency of Harmful Algal Blooms	9	11	41	95	32	10	11	222	431
The supply of clean drinking water	11	20	73	118	94	72	33	10	431

**TABLE 4:** Distribution of answers among respondents for future expectations with the following choices: much worse = −3, slightly worse = −2, worse = −1, the same = 0, better = 1, slightly better = 2, much better = 3, and prefer not to answer = 99.

Compared to 10 years ago (2009) would you say the state of the following features in Palawan is better, worse the same?	−3	−2	−1	0	1	2	3	99	Total
Amount (number) of wild fish, diversity of fish types	87	82	83	53	45	36	19	26	431
Amount of wild shellfish, diversity of shellfish types	65	64	72	92	44	28	23	43	431
Amount of fish aquaculture (e.g., fish cages)	27	27	38	50	31	32	16	210	431
Amount of shellfish aquaculture (e.g., mussel lines)	9	8	13	40	11	20	15	315	431
Amount of seaweed farming	7	5	14	33	22	32	22	296	431
Quality of coral reefs, diversity of coral types	56	46	70	57	64	45	22	71	431
Seagrass coverage, number of seagrass species	27	23	48	108	57	62	38	68	431
Mangrove coverage, diversity of mangrove types	22	34	39	70	70	106	55	35	431
Other beach tree cover	28	12	39	123	79	77	39	34	431
Amount of farming pesticides/fertilizers in the water	21	25	62	82	27	26	12	176	431
Amount/concentration of sewage in the water	39	43	92	81	41	29	14	92	431
The amount of plastics/rubbish in the water	51	54	84	79	52	46	34	31	431
The color and smell of the sea water	32	37	72	165	33	47	15	30	431
The taste of fish/shellfish from these waters	9	18	40	268	23	32	12	29	431
The frequency of Harmful Algal Blooms	13	14	56	79	20	12	11	226	431
The supply of clean drinking water	18	18	29	111	91	76	42	46	431

### Survey

The issues relevant for this study covered three main areas: habitats (mangrove, seagrass, and coral reef), provisioning ecosystem services (fisheries, aquacultures, shellfish, and seaweed farming), and risk factors [plastic, seawater quality, pesticides, and harmful algal blooms (HABs)] (see Tables 3, 4 for an overview of the questions, responses, and response scales). In addition to these questions about these topics, demographic variables (age, gender, income, education, religiosity, and area of residence) were collected. Between June 2018 and May 2019 necessary permits and agreements were secured (permit and memorandum of agreement with barangays and municipal governments). Since the study is a part of international collaboration including the Universities of Exeter and Plymouth in the United Kingdom, ethical approval was sought from and granted by the following: (a) the University of Exeter Medical School Research Ethics Committee (approval reference: May 19/B/185) and (b) the National Ethics Committee of the Department of Science and Technology in the Philippines (NEC Code: 2019-002-Creencia-Blue). To assure that the information would be understood by respondents, pilot testing was done and necessary adjustments were made such as shifting from self-administered questionnaire to face to face interview, redesigning the information, and reducing the number of questions.

The survey was then administered among the selected barangays in each municipality. Due to expected differences in the levels of education among the respondents, an orally administered questionnaire loaded on tablet computers (Samsung Galaxy Tab A) with free data collection software (kobo v. 2) was utilized. Questionnaires (see Tables 3, 4) were offered in both, English and Filipino (the national language of Philippines) which could be switched depending on the preferences of the respondents. Specifically, trained research assistants visited each household of the 431 participating coastal residents over the period of June and July 2019. Due to some difficulties in accessing coastal communities and for security reasons, interviewers relied on local guide from the barangays to select the respondents with some conditions to achieve relatively scattered respondents across the area while assuring the safety of all interviewers. Although interviews lasted for up to 90 min in total, only approximately 5 min of this were spent on the questions relevant for this study; the other questions were part of the wider Blue Communities research. A token was given to the interviewee at the end of the interview in the form of small gifts (e.g., tumbler) as a sign of appreciation and gratitude.

### Data Analysis Strategy

Initial analysis in R was then performed for data screening and quality checks. Removal of outliers of perceptions for all issues was done using boxplot analysis. Then the normal distribution of data was checked using a frequency distribution graph with skewness calculated. The skewness of perception across all issues ranged from −0.605 to 0.632.

To determine if the present perceptions were different compared to 10 years ago, one-sample *t*-tests against zero (a score of zero indicates they have stayed the same) were used. Subsequently, a one-way ANOVA was used to determine differences of perceptions across demographics (gender, age, location, income, and education). This was followed by using Dunnett’s T3 test showing significant group differences. Effect size was also calculated using Cohen’s D in R (rstatix package). The same process was repeated for the future expectation data across issues.

## Results

### Environmental Quality Perceptions: Present Compared to 10 Years Ago

**FIGURE 2:**
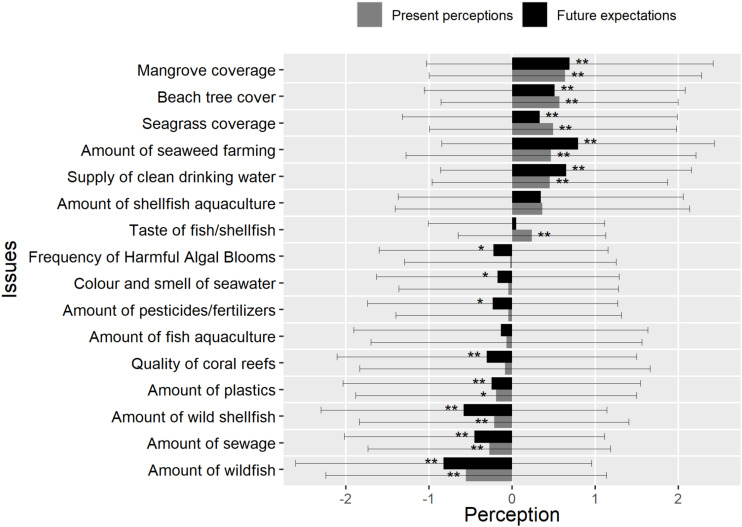
Response of respondents to questions, “What do you think of the following compared to 10 years ago?” (Present perception) and “What do you think of the following in the next 10 years compared to now?” (Future expectation) with the following options (the *x* axis): –3 is much worse, –2 is worse, –1 is slightly worse, 0 is the same, 1 is slightly better, 2 is better, and 3 is much better. Issues with ** are areas where high significant differences were observed (*p* < 0.01). Issues with * are areas where significant differences were observed (*p* < 0.05).

The one-sample *t*-tests revealed that some issues were perceived to be better than 10 years ago; some were perceived to be worse and some remained the same (no statistically significant difference to zero). Mangrove coverage, beach tree cover, seagrass coverage, amount of seaweed farming, supply of clean drinking water, amount of shellfish aquaculture, and taste of fish or shellfish were perceived to be significantly better compared to 10 years ago (*ps* < 0.05), but the magnitude of perceived change was small to medium (Cohen’s D range for difference from zero: 0.242 to 0.644) (Figure 2). Among these issues, mangrove coverage was perceived as having the most positive trend compared to 10 years ago, but the effect size was between small and medium (Cohen’s *D* = 0.39), followed by beach tree cover (Cohen’s *D* = 0.40) and seagrass coverage, (Cohen’s *D* = 0.33), then the amount of seaweed farming, supply of clean drinking water, amount of shellfish aquaculture, and taste of fish or shellfish which have all small effect sizes ranging from 0.20 to 0.34. No changes were perceived for frequency of HABs, color and smell of seawater, amount of pesticide or fertilizers, amount of fish aquaculture, and quality of coral reefs when the respondents compared these issues to their quality from 10 years ago (*ps* > 0.05). The amount of plastic, amount of wild shellfish, amount of sewage, and the amount of wild fish were perceived to be significantly worse than 10 years ago (*ps* < 0.05) although the perception of change was small to medium as well (Cohen’s D range from −0.551 to −0.33). Among the issues that were perceived worse than 10 years ago were the amount of wild fish though not really noticeable (Cohen’s *D* = 0.33), followed by an increase of sewage (Cohen’s *D* = 0.19) and a decrease of wild shellfish (Cohen’s *D* = 0.13), respectively. It is also worth noting that SDs for all issues were large (up to ±2 SD), indicating considerable variation in the perceptions of the respondents.

### Environmental Change Expectations: Future Compared to Present

When asked about expected changes regarding the same issues in the future, respondents expected mangroves coverage, beach tree cover, seagrass coverage, amount of seaweed farming, supply of clean drinking water, amount of shellfish aquaculture, and taste of fish or shellfish to get better compared to the present (*ps* < 0.05) (Figure 2). Among these issues, the amount of seaweed farming was expected to increase the most with a medium effect size (Cohen’s *D* = 0.48), followed by mangrove coverage (Cohen’s *D* = 0.40) and supply of clean drinking water (Cohen’s *D* = 0.43). The amount of aquaculture was expected to remain the same (*p* > 0.05). The color and smell of seawater, amount of pesticide or fertilizers, frequency of HABs, the amount of plastic, quality of coral reefs, amount of sewage, the amount of wild shellfish, and the amount of wild fish were expected to get worse in the future compared to the present (*ps* < 0.05), with varying effect sizes (Cohen’s D ranged from −0.550 to −0.195). Among these issues, the most negative expectations were found for the amount of wild fish with a relatively moderate effect size (Cohen’s *D* = 0.40) followed by the amount of wild shellfish (Cohen’s *D* = 0.34) and the amount of sewage (Cohen’s *D* = 0.56). It is also worth noting that SDs for all issues were larger than for the present-compared-to-past perceptions (range: ±2 to ±3 SD), indicating greater uncertainty about the future.

### Demographic Differences in Perception

Generally, perceptions across different demographic groupings (gender, age, education, income, and location) were relatively similar for perceptions of the present and the future with only few differences.

For present-compared-to-past perceptions, respondents in different education and income categories had similar perceptions of all issues (*ps* > 0.05) (Supplementary Figures 2, 3), but visual inspection of the means indicates that the two high income groups (656.09–1,640.26 and 1,640.26–2,460.40) tended to view issues more negatively than other groups; however, it is to be noted that only few respondents were represented this group (*n* = 4 and *n* = 2, respectively); hence, we applied a non-parametric Kruskal–Wallis test for this particular instance. While respondents varying in age and gender showed varying perceptions in a few issues (Supplementary Figures 4, 5), though not statistically significant, in general, males tended to have more extreme perceptions than women. The present situation compared to 10 years ago, was perceived significantly different by respondents belonging to different age groups on the subject of clean drinking water supply (*p* < 0.05) (Supplementary Figure 4). The younger age cluster (18–29) expressed opposing perceptions compared to the oldest age cluster (60–90), in terms of the young people perceiving the drinking water quality to remain the same whereas the older people perceived it to improve, with a moderate effect size (Cohen’s *D* = 0.53). Men and women showed differences in the perceived frequency of HABs compared to 10 years ago (*p* < 0.05) (Supplementary Figure 5). Male respondents tended to perceive this issue getting slightly better (less HABs) while female respondents perceived the issue to get worse (more HABs), though the effects were relatively small (Cohen’s *D* = 0.35).

For future expectations, the respondents had similar expectations across genders and income clusters (*p* > 0.05) with regard to all issues (Supplementary Figures 6, 7). Respondents belonging to different age and education had varying perceptions but only with regard to a few issues (Supplementary Figures 8, 9). In particular, respondents in different age groups had significantly different expectations (*p* < 0.05) of clean drinking water supply (Supplementary Figure 8). Though all age groups expected better quality in the future, the youngest (18–29) and the oldest age group (60–69) differed significantly (*p* < 0.05) with the latter having more positive expectation than the former. Respondents in different education categories also had significantly different perceptions (*p* < 0.05) on the supply of clean drinking water, color and smell of seawater, amount of pesticide or fertilizers, and the amount of plastics (Supplementary Figure 9). In all of these issues, except the amount of plastics, respondents with high school level of education expressed lower or opposite expectations compared to respondents with elementary education (*p* < 0.05). Respondents with college level of education had more negative expectations on the amount of plastics than respondents with elementary level of education (*p* < 0.05).

**FIGURE 3:**
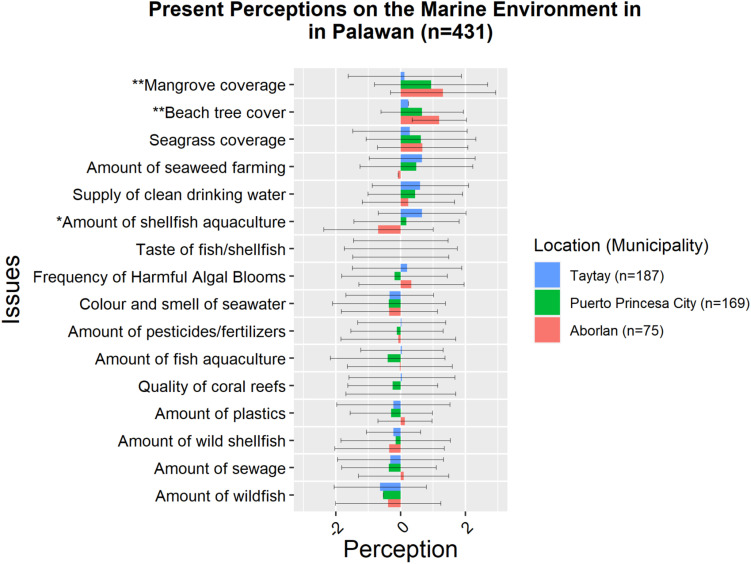
Present perceptions across different locations. Issues with ** are areas where high significant differences were observed (*p* < 0.01). Issues with * are areas where significant differences were observed (*p* < 0.05).

**FIGURE 4:**
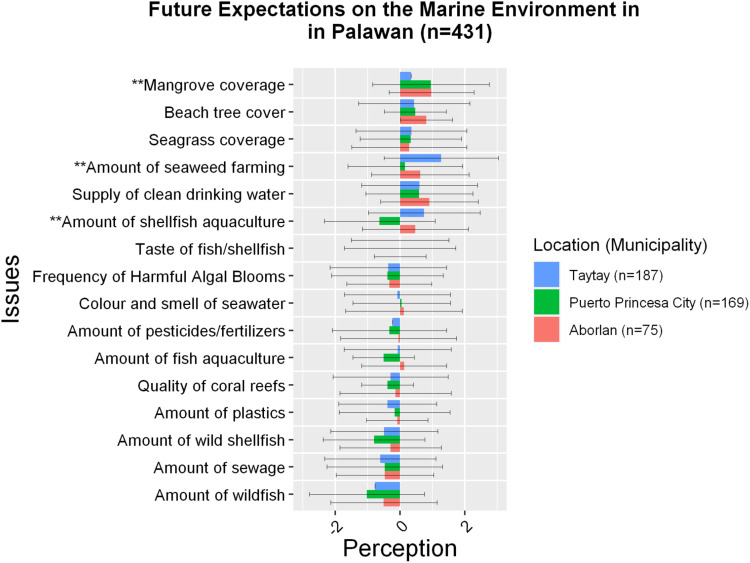
Future expectations across different locations. Issues with ** are areas where high significant differences were observed (*p* < 0.01). Issues with * are areas where significant differences were observed (*p* < 0.05).

The most significant differences were identified between people from different areas of residence – Aborlan, Taytay, and Puerto Princesa City. Coastal communities in Aborlan perceived that mangrove coverage and beach tree cover to have largely improved in the past 10 years and expected to improve further in the next 10 years. Coastal communities in Taytay perceived significantly less improvement with regard to mangroves coverage (*p* < 0.05) (Figure 3). This effect was large (Cohen’s *D* = 0.74). Meanwhile, seaweed farming was expected by the respondents in Taytay to have a positive future as compared to people from Puerto Princesa City (*p* < 0.05) and the effect was moderate (Cohen’s *D* = 0.70). Moreover, the amount of shellfish aquaculture was expected by the people in Taytay and Aborlan to improve in the future while those in Puerto Princesa City expected shellfish aquaculture to become worse (*p* < 0.05) (Figure 4). All the three calculated effect sizes were large between Taytay and Puerto Princesa City (Cohen’s *D* = 0.82) as well as between Aborlan and Puerto Princesa (Cohen’s *D* = 0.76). All municipalities tended to perceive the frequency of HABs differently within the past 10 years although these differences did not reach significant level. Different perceptions were also identified between municipalities for beach tree cover, pointing toward an improvement. People in Aborlan perceived significantly more improvement in their beach tree cover than those in Taytay (*p* < 0.05), but only a small effect was observed (Cohen’s *D* = 0.24).

## Discussion

The study identified the perceptions of community members from the city of Puerto Princesa and two selected municipalities (Aborlan and Taytay) in the province of Palawan, Philippines of their local marine environment quality over time. Although strenuous attempts at more participatory decision-making are made (both in research and practice), illegal resource use and extraction still exist.

Our stance is that involving the local community more in effective coastal resource management is crucial, as they are the ones who directly depend on it. Therefore, knowing how the local communities perceive their marine environment is essential for planning, communicating, and working together toward sustainable resource management. A mismatch between community perceptions and governmental regulations can be the source of severe miscommunication among resource managers and resource users, and a subsequent failure of conservation effort (Gelcich and O’Keeffe, 2016). There is evidence that MPAs implemented with a top-down approach and without participation of local stakeholders and communities are at risk of becoming “paper” parks (Rife et al., 2013; Advani et al., 2015; Pieraccini et al., 2017). In this work, relevant marine environmental issues were co-identified and subsequently measured with regard to current perceptions of environmental quality, as compared to the past (10 years ago) as well as future expectations (in the span of 10 years).

### Present Perceptions and Future Expectations

Overall, present and future perceptions of the locals across Palawan show a diverse picture of positive and negative trends. Coastal communities indicate that they perceive a positive development in mangrove and seagrass ecosystems as well as in the aquaculture and seaweed sector. At the same time, they recognize environmental degradation and decline in some resources, such as wild fish and wild shellfish. Pollution-related issues, such as the amount of pesticides and fertilizer entering the environment, plastics litter, and sewage pollution was consistently perceived as showing a negative trend from the past into the future.

The expectations of the locals for improvements in seaweed farming and shellfish aquaculture support the global projection, that by 2030, 53% of the marine protein production will be from aquaculture (FAO, 2020), and this is also in line with local initiatives. All three municipalities, Puerto Princesa City, Aborlan, and Taytay Palawan are beneficiaries of the livelihood support by the local government units for seaweed farmers. Seaweed farming represents the major livelihood in Palawan, and ranks number one in seaweed production in the entire Philippine archipelago.

The perception that fish stocks declined over the last decade and the expectation that this trend will continue in the future is backed up by the most recent report by the FAO (2020) describing the struggles of capture fisheries globally. In the Philippines, the volume of production from commercial fisheries in 2019 went down by 1.77% in 2017. This subsector comprised 45.29% of the total capture fisheries output (Philippine Statistics Authority [PSA], 2020).

The perceptions of increasing environmental pressure through pollution is in line with other studies showing that pollution from farming, household, and fishing is consistently being perceived as severe threats to the marine environment (Potts et al., 2016; Tonin and Lucaroni, 2017; Gkargkavouzi et al., 2020). Compared to other issues, pollution is a well-known and visible topic among the general public, because the problem receives considerable attention by the media and conservation groups (Lotze et al., 2018).

We also identified some areas in which the perceptions of the reported community are not fully in line with the observed behavior and related policies. As an example, plastic pollution is perceived to become more serious; however, this does not translate into the behavior of the people yet. Potential reasons could be that communities do not experience the consequences of plastic pollutions just yet or other areas of living have a higher priority. Another example is illegal fishing. Wild fish stock is perceived and expected to decline. One could consequently expect adherence to policies protecting the resource fish in the long terms, such as bans of destructive fishing methods. Instead, fishermen are still engaged in these activities, which could be explained by the common dilemma theory around limited resources (Gifford and Wells, 1991). Illustrating these cases shows that considering the perception of the people can be helpful for tailored communication approaches and education programs. Actively involving communities in responsible resource management can increase policy adherence and reinforce loyalty to regulations.

Since perceptions of respondents in this study varied across issues, different approaches are necessary for the management of different resources and habitats. Depending on individual, trends of perceptions in terms of positive, negative, or relatively neutral, different mechanisms are being activated. Anticipated negative events or consequences have been found to evoke pro-environmental intentions (Sanna, 1996; Carrus et al., 2008; Pestridge, 2017). Several studies point toward fear or worries related to the future being a principal antecedent of risk prevention behavior, given a reasonable level of self-efficacy, a sense of personal control over the issue (LaTour and Tanner, 2003; Dillard and Anderson, 2004). However, in case of low self-efficacy agency levels, negative perceptions and anticipations lead to the feeling of helplessness, rejection of information, or maladaptive responses. If people feel that they do not have the ability to respond to the fear appeal appropriately (i.e., danger control), threatening information can lead to maladaptive responses (i.e., fear control) (Witte, 1994). Anticipated positive events or consequences have the potential to encourage pro-environmental behavior, because people who are optimistic about the future undertake more preparation actions (Ojala, 2012; Schaffner et al., 2015; Brieger, 2018). Imagining a positive future can increase the likelihood of future to become reality if people have a high level of self-efficacy (Adriaanse et al., 2010; Bain et al., 2015). Participatory involvement has the potential to increase a sense of control over local environments and should be beneficial, regardless of positive or negative perceptions.

We would like to point out that the perceptions of the present as compared to the past and expectations for the future were highly correlated (*p* < 0.001) (Supplementary Figure 10). Issues perceived as positive in the present relative to the past tended to be perceived as positive in the future. Issues that were perceived as negative in the present tended to be perceived as negative in the future. This can be explained by psychological processes concerning the past and the future being intertwined (Pahl et al., 2014). Memories of the past are often used to simulate the future and both processes share regions in the brain (Szpunar et al., 2012).

### Demographic Differences

We did not find many significant differences between participants belonging to different demographic clusters regarding age, gender, income, and education. Out of 16 issues, only six issues display significant demographic differences in these demographic variables. More differences have been identified among the three municipalities, Puerto Princesa City, Aborlan, and Taytay.

In previous studies, fishers belonging to different age clusters were found to display varying environmental perceptions, which can be described as the shifting of the base-line syndrome (Pauly, 1995; Venkatachalam et al., 2010; Bender et al., 2013; Maia et al., 2018). However, in the previous studies, time was not clearly defined. In this study, we have limited the respondents to give their perceptions in a 10-year time frame (i.e., 10 years before and 10 years after), and only perceptions of drinking water quality differed among the age groups. Younger generations did not perceive changes in their drinking water quality as compared to elders who perceived the supply as getting better. The installation of reliable clean water supply in remote coastal areas is just under development in recent years and therefore it might mark a significant environmental change for older generations and could influence their perception on drinking water, while for younger generations, this might be the same, a potential shift in the baseline (Pauly, 1995). This contrasted with observations found in Arctic communities where perception across ages on freshwater sources through municipal supply was not different (Alessa et al., 2008). So far, this study in general did not observe changes in perceptions across age, gender, income, and education which might be due to the time limit imposed.

The Bureau of Fisheries and Aquatic Resources (BFAR), in collaboration with local government units, conducts periodic monitoring of the occurrence of HABs in Puerto Princesa City and Taytay, Palawan and posts the results in its Shellfish Bulletin (Bureau of Fisheries and Aquatic Resources [BFAR], 2021). The seasonal occurrence of HABs has been reported in coastal areas of Palawan within the past 15 years (Azanza et al., 2008). This often leads to red tide alerts, banning the gathering, shelling, and consumption of shellfish coming from these areas by the government. We found gender differences in how HABs are perceived. Women perceived the frequency of HABs to increase, while men perceived them to decrease slightly. Among the coastal residents in the Persian Gulf, Mirza Esmaeili et al. (2020) reported that the male respondents were more concerned about the occurrence of red tides than women, which is contrary to our results and was explained by men being more affected. In Palawan, those who are involved in shellfish gathering, selling, and buying are predominantly women. Consequently, women are directly affected and more informed on the advisory on these activities when a red tide alert is issued. However, we need to keep in mind, that the sample consisted of slightly more female than male respondents and treat this finding with caution.

Significantly, different future expectations of pollution-related issues (drinking water quality, fertilizer run off, and plastic pollution) between high and low educational clusters were found in the present research. This reflects a common phenomenon. In a survey over 119 countries around the world, Lee et al. (2015) investigated common predictors for climate change awareness. The only worldwide common predictor turned out to be in the educational level, indicating that groups with lower educational level have less awareness and therefore perform less adaptation and mitigation activities. Educational level determines the extent to which people have access to (written) information and therefore potentially gain the ability to question common practices, such as littering.

Differences in perceptions and expectations were identified among people from the three different areas of residence, Puerto Princesa City, Aborlan, and Taytay. As illustrated in Figure 1, the three areas are located in a relative distance to each other. While Puerto Princesa is in the middle of the Island and represents the municipality with the highest density of residence, most economic activities and the arrival point of all international tourists, Taytay is located at the northern end of the island and represents a remote area with very little, but developing tourism. Aborlan is located south of Puerto Princesa and represents an area that hosts no tourism and concentrates on fisheries as its main economic sector in the coastal areas.

Respondents in Aborlan and Puerto Princesa have a positive perception of the status of mangrove and beach tree coverage, while those in Taytay perceived these issues to decline. This difference could be attributed to the media attention and community-based efforts in Puerto Princesa regarding reforestation of mangroves (Strain et al., 2019). Puerto Princesa promotes itself as the “City in the Forest,” and has a vision to be a model city in sustainable development, ecotourism, and cleanliness in the Philippines (Jayagoda, 2015; Manalo, 2017). Among the projects that have been catching wider media attention and sparked community involvements in the past two decades are the “Feast of the Forest,” and annual tree planting project, and the “Love Affair with Nature,” a mass wedding ceremony in mangroves which culminates in mangrove tree planting in coastal areas of Puerto Princesa (Jayagoda, 2015). These activities resulted in the planting of millions of forest and mangrove trees with high survival rates. Research findings showed that the mangroves and beach forest cover around Puerto Princesa have indeed increased compared to 1992 records (Jayagoda, 2015), something that is promoted frequently in the local media. Moreover, the communities interviewed in Aborlan are located in close proximity to the Western Philippine University. The outreach activities provided by this University could have contributed in raising the knowledge and awareness of the respondents. According to Gkargkavouzi et al. (2020), coastal communities perceive research centers as competent sources for information on the management and protection of the environment. Taytay municipality is located far north of Puerto Princesa and has limited main media channel reporting about the conservation activities as compared to Puerto Princesa City where broadcasting companies are located. In Taytay, mangroves are not celebrated in the same way as they are in the capital, which might be the reason for the perceptual differences we identified.

Respondents in Taytay reported significantly more positive perceptions on shellfish aquaculture in the present and in the future compared to respondents from Aborlan or Puerto Princesa City. In contrast to the other two municipalities, shellfish aquaculture is a major source of livelihood for many coastal communities along Malampaya Sound in Taytay. The production of shellfish has increased over the recent years in Palawan, with Taytay being the main contributor to this sector. Moreover, the communities along Taytay Bay have been involved in seaweed farming of species, such as *Kappaphycus* and *Eucheuma*. Within the past 10 years, the seaweed production in the Taytay area has expanded considerably. Our results are in line with previous studies demonstrating that people have positive attitudes on the marine environment when they directly benefit from it on a personal, socio-cultural, and economic level (Hynes et al., 2014; Pearson et al., 2014; Hawkins et al., 2016).

### Limitations

Since we were not able to include actual (objective) observed changes in each environmental issue in this study, perceptions of each issue cannot be directly cross-verified with the state of the natural environment. Nevertheless, these perceptions are useful in understanding the attitudes and behavior of communities in relation to each issue (Chu et al., 2010). Depending on the perceptions of the communities, their response and acceptance to policies for each issue might vary (Gelcich et al., 2005).

Positive and negative trends observed in this study were relatively small, whereas SDs were relatively big. This indicates that albeit overall trends are identifiable, we need to be aware of inter-individual differences being relatively large. This can be explained by the nature of the data. The perceptions of the people’s can be influenced by daily mood or personal differences, such as an optimistic or pessimistic disposition regarding the future. Further, perceptions are not an accurate measurement of environmental quality, but an indicator of how this environmental quality is perceived by people. Especially when a time dimension is added and people are asked to indicate their perceptions and expectations of environmental changes over time, responses will differ between participants due to their individual life story as well as temporal biases. The further away an event lies in the future, the more unsure are people about its outcome, as explained in Pahl et al. (2014), which can explain why the SDs for future expectations are larger than the ones for present perceptions.

## Conclusion and Policy Implications

In this work, we identified 16 relevant marine environmental issues in Palawan and investigated how coastal communities perceive these issues changing across time, from past to future. We showed that coastal communities in Palawan perceive many issues to be worse today as compared to 10 years ago, while other issues are perceived to improve. The amount of wild fish and shellfish, and pollution-related issues, such as plastics, sewage, fertilizer/pesticide, and harmful algal bloom were all perceived to follow a negative trend. However, the amount of mangroves, seagrass and beach tree coverage, and seaweed farming and the supply of clean drinking water were perceived to follow a positive trend over the last 10 years up to now. A strong correlation exists between the present perception and future expectation, which suggests that the way the marine environment is perceived at present directly affects how things are expected to develop in the future. The perception of the issues was not much affected by demographic factors, such as age, gender, education, and income. However, differences were found in perceptions between municipalities, indicating that things are perceived differently depending on the direct geographical context.

For successful marine management, policy decisions should consider the perspectives of the local community. Efforts are underway to use community consultations, but there is room for improvement. The results of this study enhance our knowledge and understanding on how local communities perceive their environment. We identified which marine environmental issues are perceived as following positive or negative trends and which issues are expected to follow positive or negative trends in the future, using a time frame of ±10 years. These insights can help inform resource management strategies and communication approaches for policy makers at the provincial and municipal levels, local government unit leaders, conservation practitioners, and other stakeholders in Palawan. In recognizing how communities perceive their environment and how they expect it to change in the future, policymakers can develop strategies that align with public views, needs and priorities, and consequently increase public acceptance and support.

## Data Availability Statement

The original contributions presented in the study are included in the article/ Supplementary Material, further inquiries can be directed to the corresponding author.

## Ethics Statement

The studies involving human participants were reviewed and approved by the National Ethics Committee of the Department of Science and Technology (NEC Code: 2019-002-Creencia-Blue) and University of Exeter Medical School Research Ethics Committee (approval reference: May19/B/185). The participants provided their written informed consent to participate in this study.

## Author Contributions

JS: data collection, development and design of methodology, formal analysis, writing—original draft, and visualization. IR: conceptualization, writing—review, editing, formal analysis, development, design of methodology, and supervision. AA and SP: conceptualization, writing—review and editing, and supervision. HB: writing—review and editing. LC: data collection, conceptualization, writing—review, and supervision. All authors contributed to the article and approved the submitted version.

## Conflict of Interest

The authors declare that the research was conducted in the absence of any commercial or financial relationships that could be construed as a potential conflict of interest.

## Publisher’s Note

All claims expressed in this article are solely those of the authors and do not necessarily represent those of their affiliated organizations, or those of the publisher, the editors and the reviewers. Any product that may be evaluated in this article, or claim that may be made by its manufacturer, is not guaranteed or endorsed by the publisher.
