# Supplementary material for: Ask the Locals: A Community-Informed Analysis of Perceived Marine Environment Quality Over Time in Palawan, Philippines
Source: Front Psychol. 2021 Aug 10;12:661810. doi: 10.3389/fpsyg.2021.661810 (PMC8382879; doi:10.3389/fpsyg.2021.661810)

Supplementary Material

Ask the Locals:

A Community-Informed Analysis of Perceived Marine Environment Quality over Time in Palawan, Philippines

Joel D.C. Sumeldan[1*], Isabel Richter[2], Arlene L. Avillanosa[1], Hernando P. Bacosa[1,3], Lota A. Creencia[1], Sabine Pahl[2,4]

[1]College of Fisheries and Aquatic Sciences, Western Philippines University, Puerto Princesa City, Palawan, Philippines
[2]School of Psychology, University of Plymouth, Drake Circus, Plymouth, Devon, United Kingdom
[3]Department of Biological Sciences, Mindanao State University-Iligan Institute of Technology, Iligan, Philippines
[4]Urban and Environmental Psychology Group, Institute for the Psychology of Cognition, Emotion and Methods, Faculty of Psychology, University of Vienna, Austria

Figure S1. Hand drawn maps from Focus Group Discussion in Puerto Princesa City

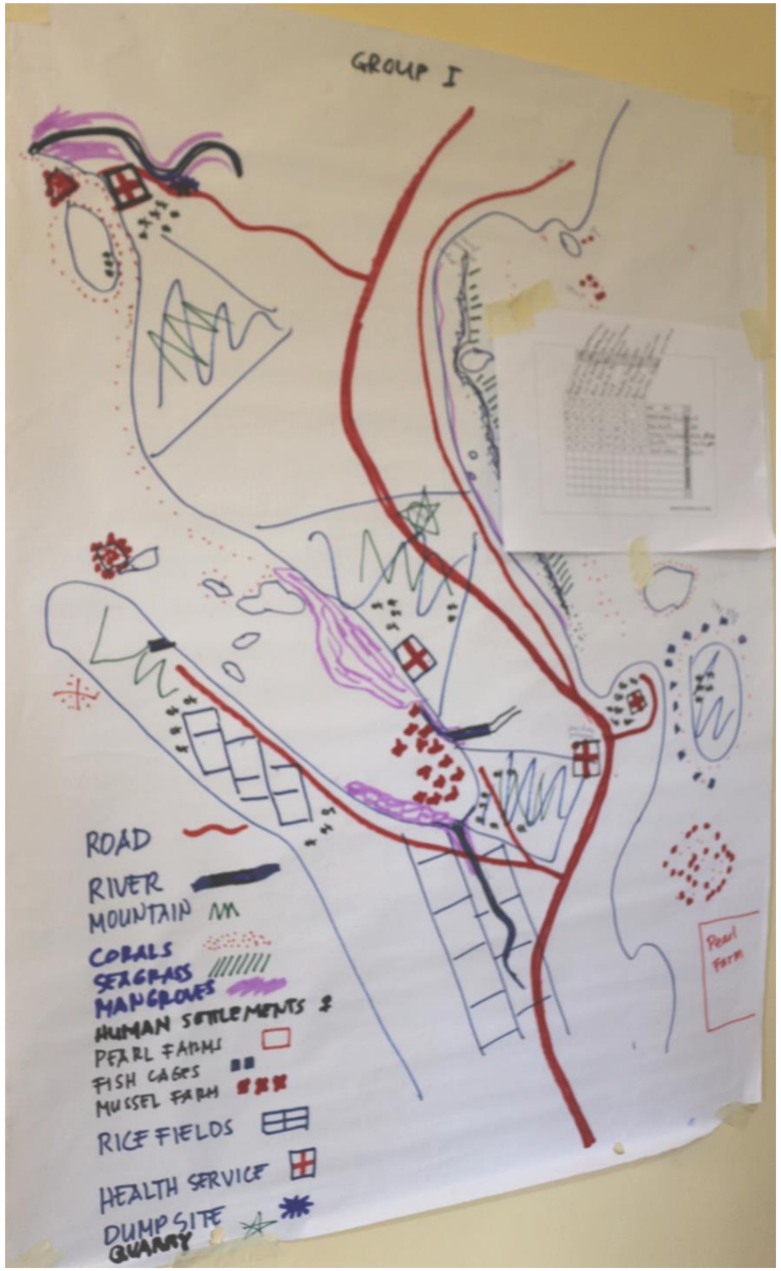

Figure S2. Present perceptions across different education. Issues with ** are where high significant differences were observed ($p<0.01$). Issues with * are where significant differences were observed ($p<0.05$)

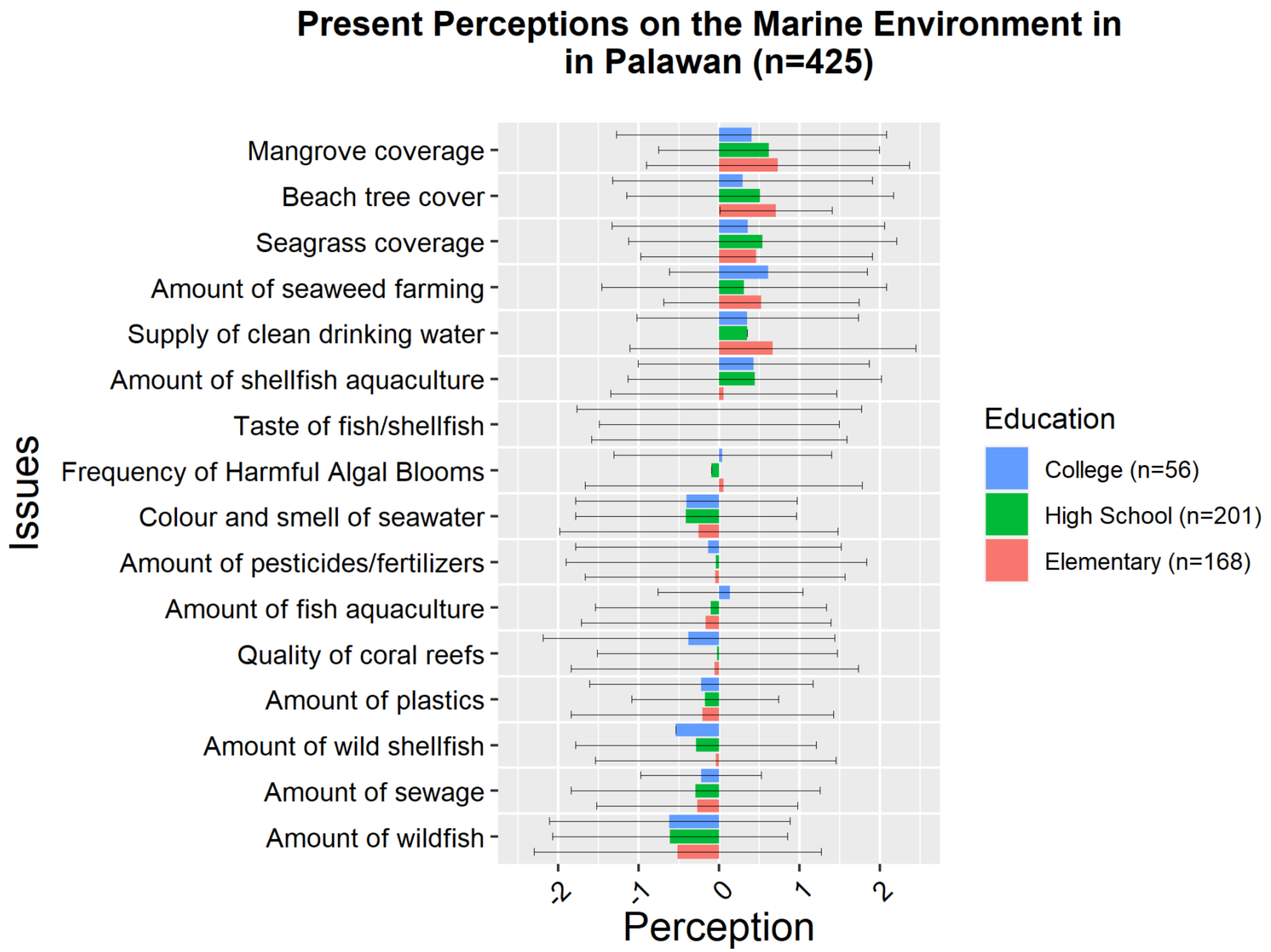

Figure S3. Present perceptions across different Income. Issues with ** are where high significant differences were observed ($p<0.01$). Issues with * are where significant differences were observed ($p<0.05$)

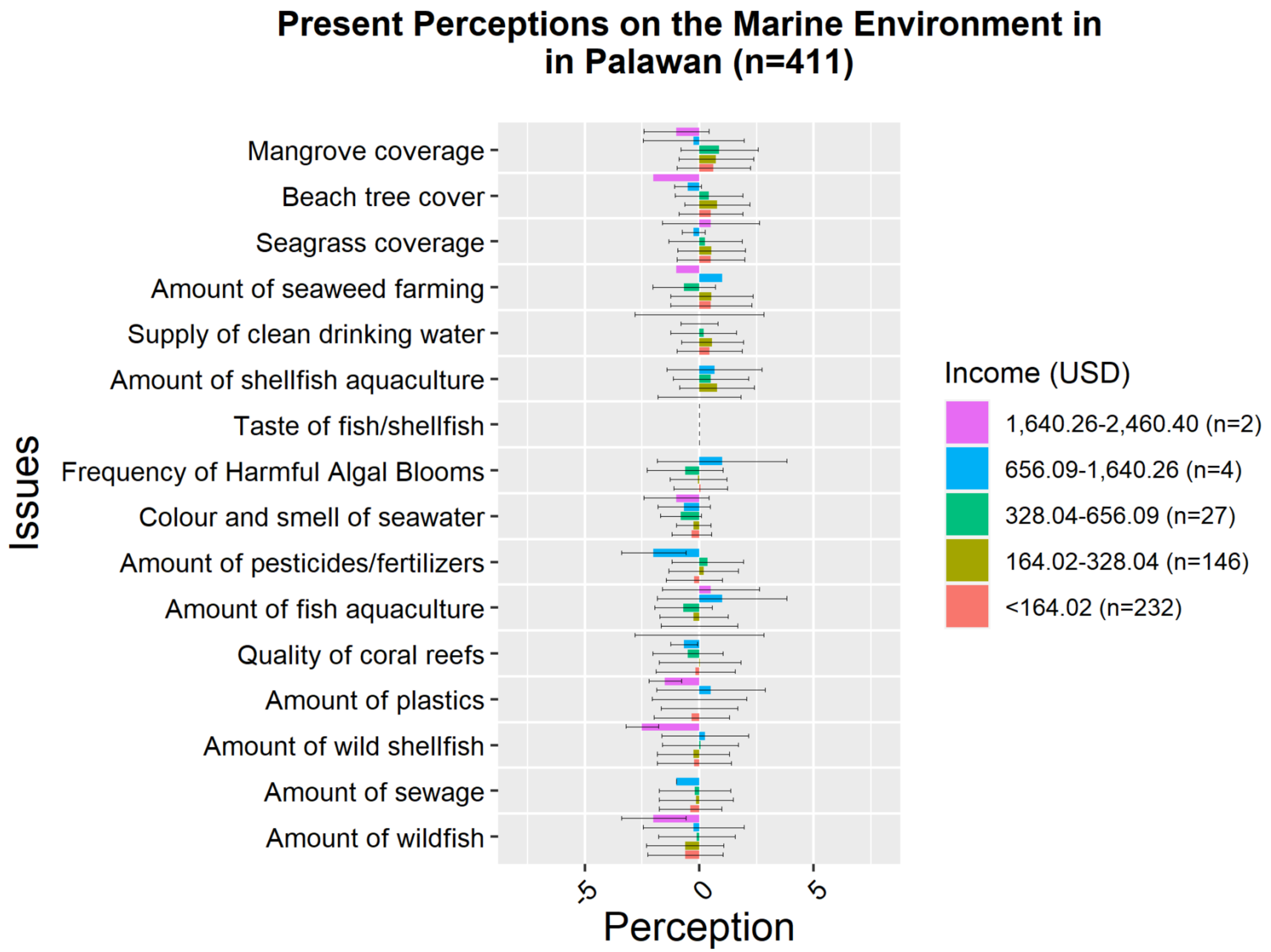

Figure S4. Present perceptions across different ages. Issues with ** are where high significant differences were observed (*p*<*0*.01). Issues with * are where significant differences were observed (*p*<*0*.05)

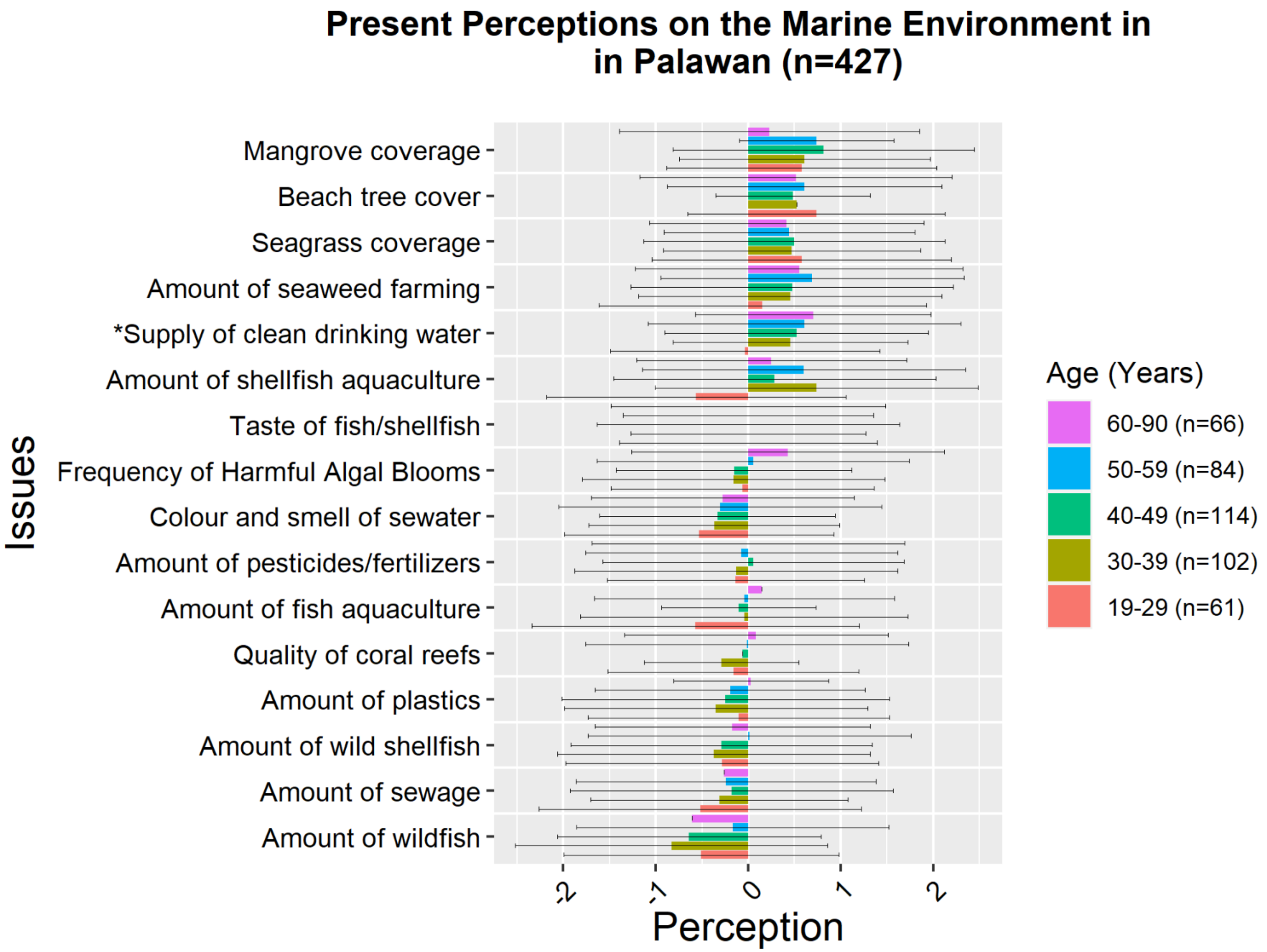

Figure S5. Present perceptions between different gender. Issues with ** are where high significant differences were observed ($p<0.01$). Issues with * are where significant differences were observed ($p<0.05$)

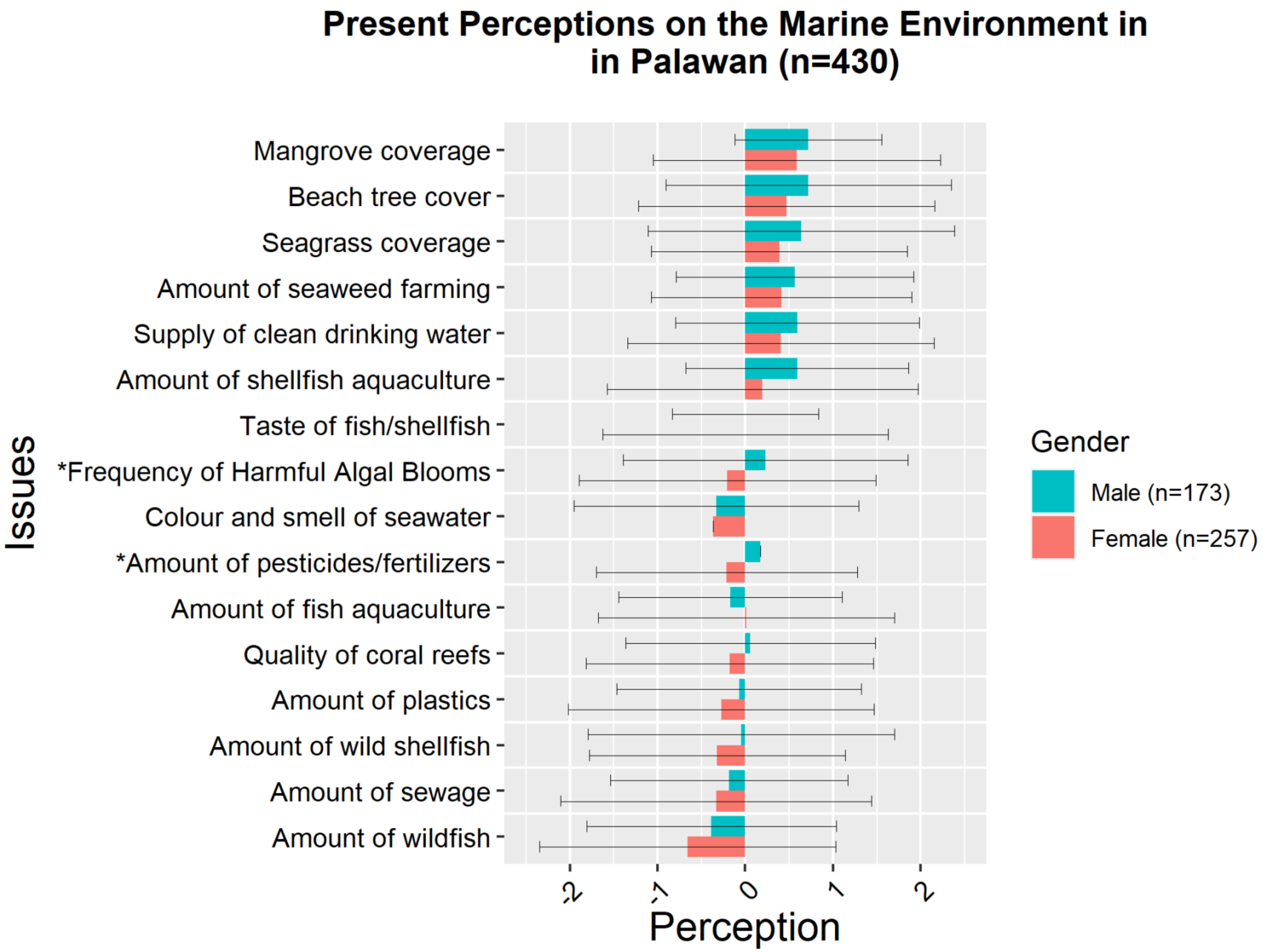

Figure S6. Future expectations between different gender. Issues with ** are where high significant differences were observed (*p*<*0*.01). Issues with * are where significant differences were observed (*p*<*0*.05)

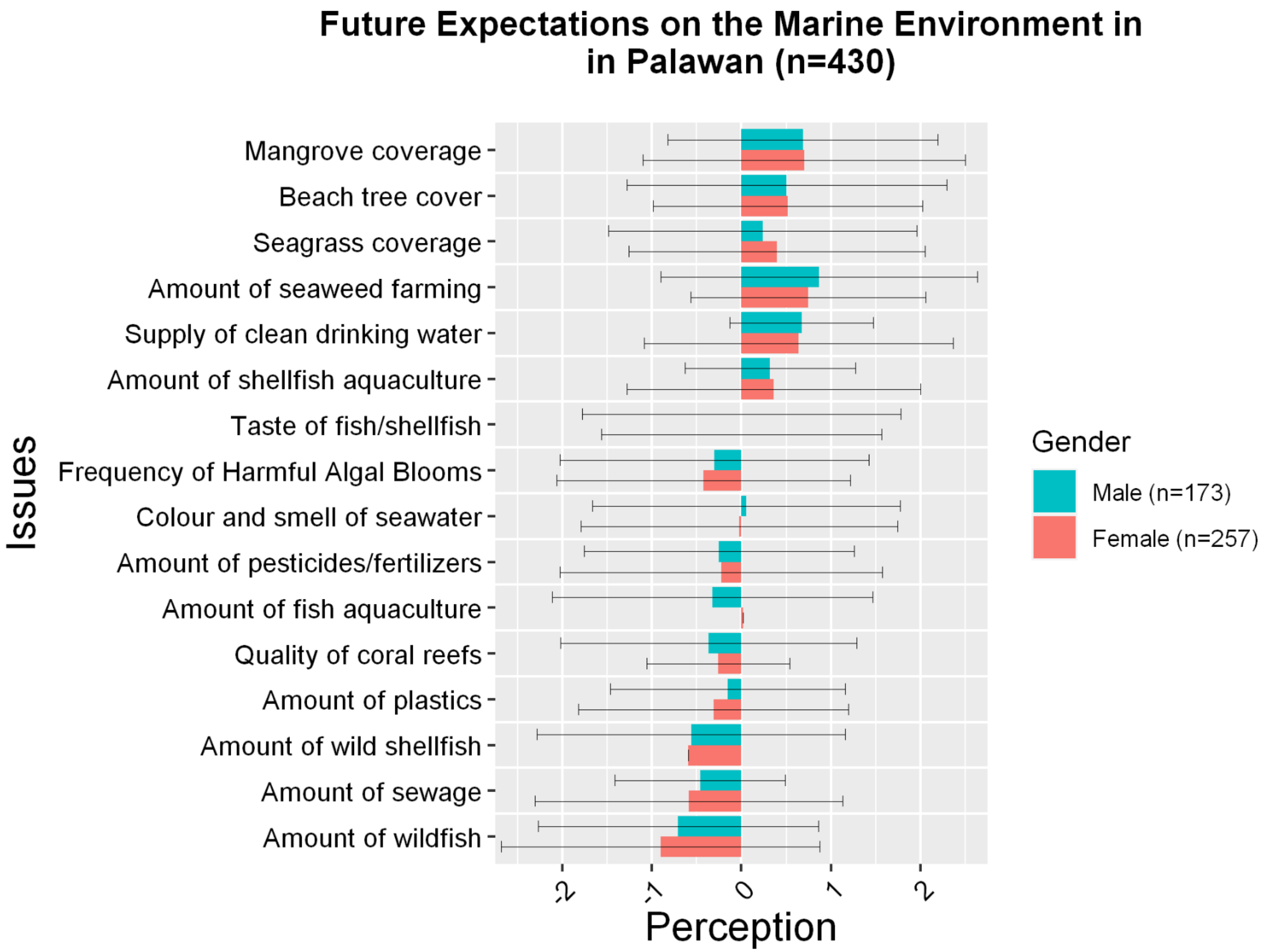

Figure S7. Future expectations across different Income. Issues with ** are where high significant differences were observed (*p*<*0.*01). Issues with * are where significant differences were observed (*p*<*0.*05)

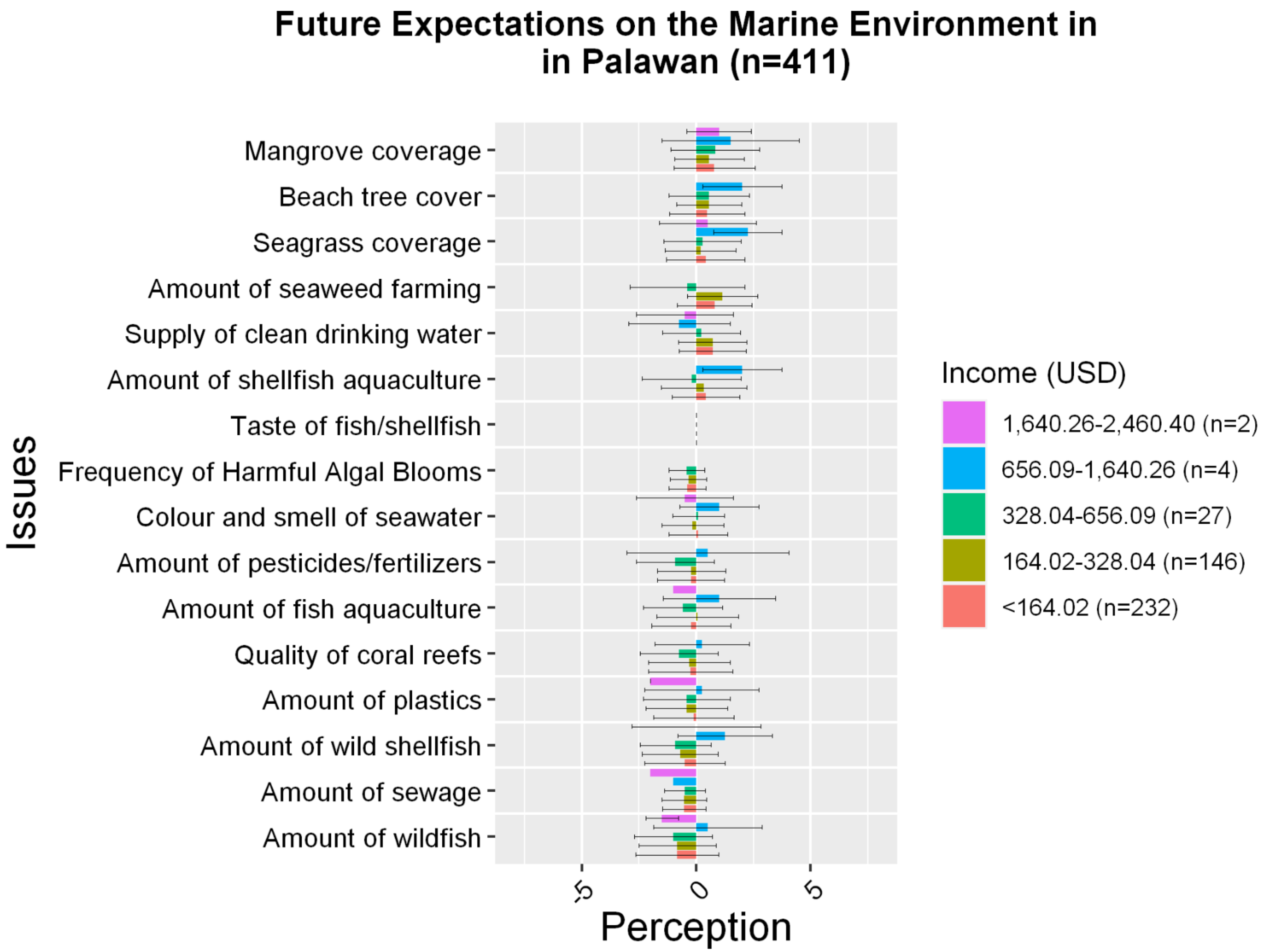

Figure S8. Future expectations across different age. Issues with ** are where high significant differences were observed (*p<0.*01). Issues with * are where significant differences were observed (*p<0.*05)

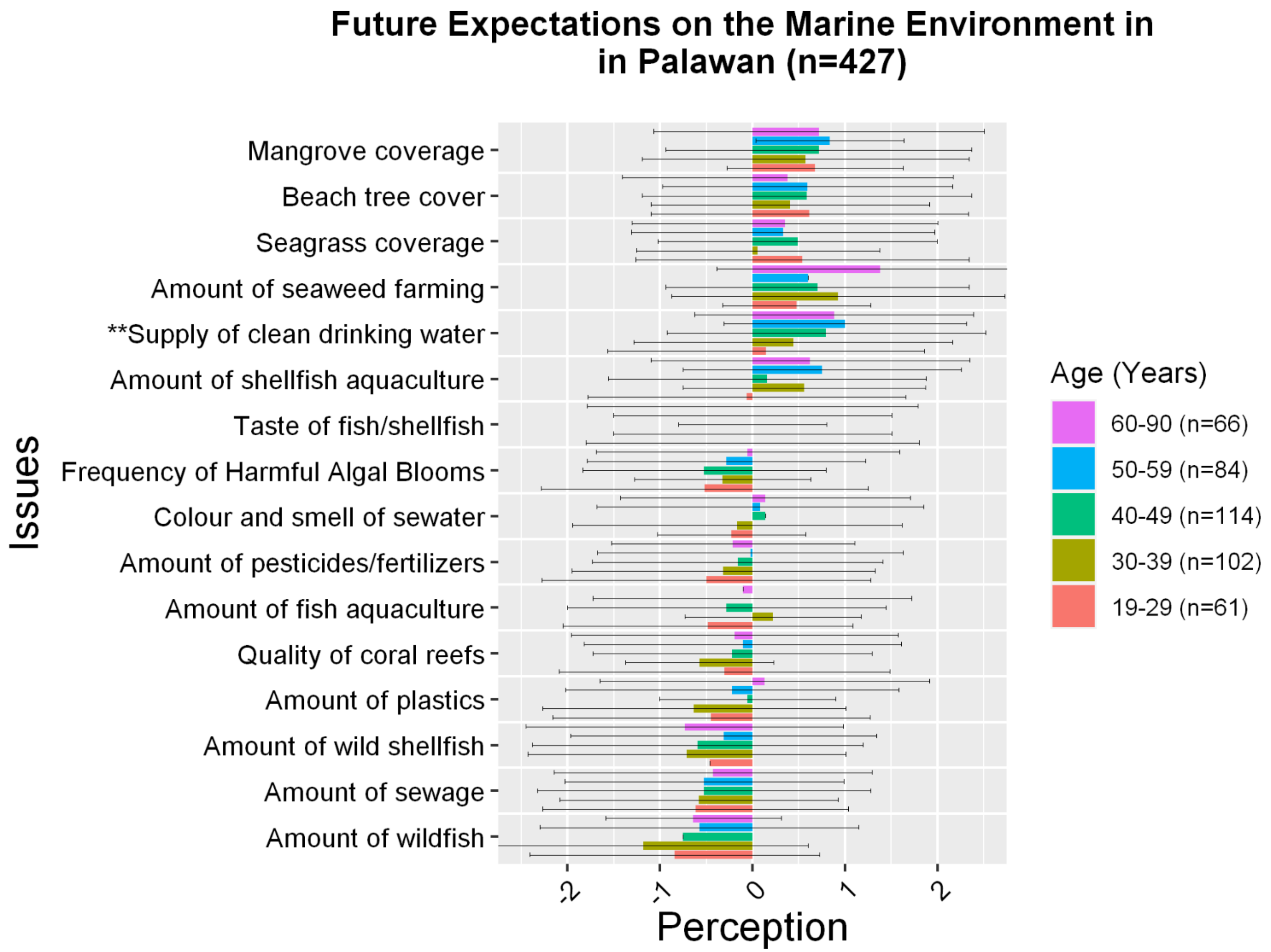

Figure S9. Future expectations across different education. Issues with ** are where high significant differences were observed (*p<0.*01). Issues with * are where significant differences were observed (*p<0.*05)

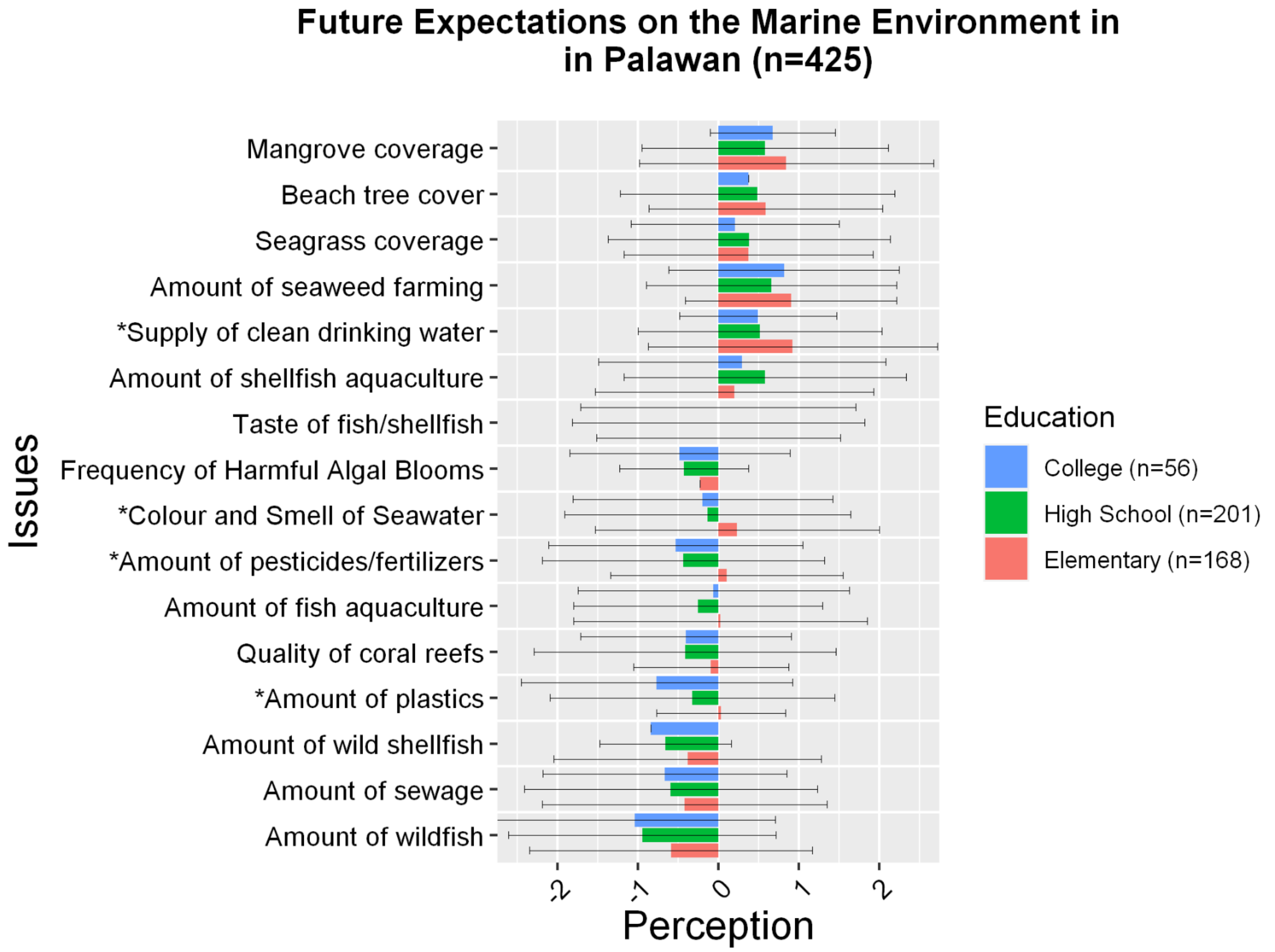

Figure S10. Correlation of present perception and future expectation

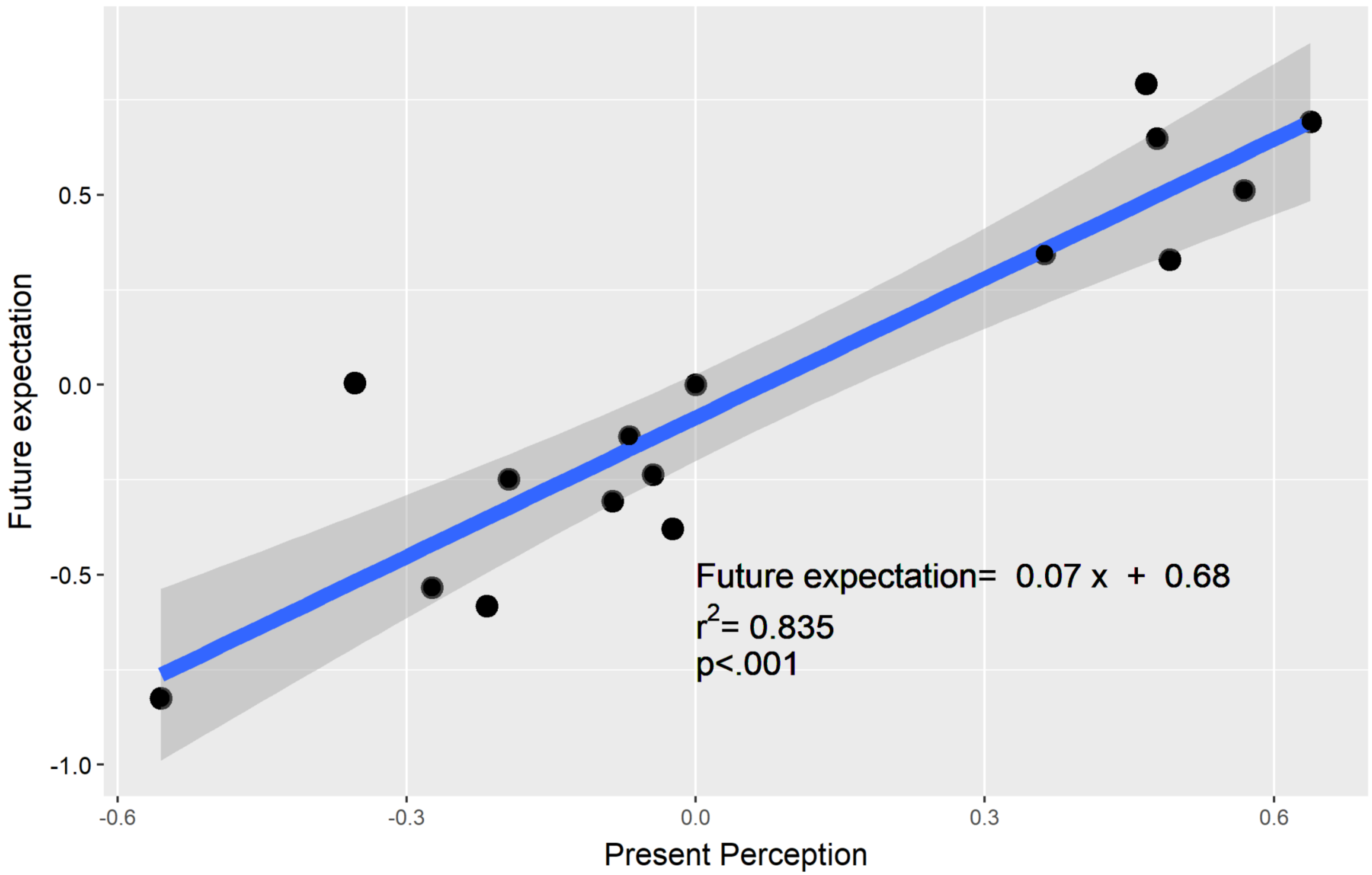

Supplement: Supplementary file 1 [file Data_Sheet_1.pdf]
